# The effect of the combined system of hydrodynamic cavitation, ozone, and hydrogen peroxide on chlorophyll a and organic substances removal in the raw water

**DOI:** 10.1038/s41598-023-37167-0

**Published:** 2023-06-21

**Authors:** Reza Shokoohi, Alireza Rahmani, Ghorban Asgari, Maysam Ashrafi, Esmaeil Ghahramani

**Affiliations:** 1 Department of Environmental Health Engineering, School of Public Health, Research Centre for Health Sciences, Hamadan University of Medical Sciences, Hamadan, Iran; 2 Department of Chemistry, University of Kurdistan, Sanandaj, Kurdistan Iran; 3 Research Institute for Health Department, Environmental Health Research Center, Kurdistan University of Medical Sciences, Sanandaj, Iran

**Keywords:** Ecology, Environmental sciences, Engineering

## Abstract

Increased levels of nutrients and algae can cause drinking water problems in communities. Harmful algal blooms affect humans, fish, marine mammals, birds, and other animals. In the present study, we investigated the use of a combined system [Hydrodynamic Cavitation, Ozone (O_3_), and Hydrogen Peroxide (H_2_O_2_)] on the removal of Chlorophyll a and Organic substances in the raw water was investigated. The Effect of different operating conditions such as pH, cavitation time, pressure, distance, flow rate, ozone dose, and hydrogen peroxide concentration was studied. Utilizing the Taguchi design method, experiments were planned and optimized. The combined system treatment yielded a maximum reduction in Chlorophyll a and Total Organic Carbon (TOC) at an optimum condition of pH 5, cavitation pressure 5 bar, flow rate of 1 m^3^/h, a distance of 25 cm from the orifice plate, O_3_ 3 g/h and 2 g/l of H_2_O_2_ concentrations. The most efficient factor in the degradation of TOC and Chlorophyll a, was cavitation pressure based on the percentage contributions of each parameter (38.64 percent and 35.05 percent, respectively). H_2_O_2_ was found to have the most negligible impact on degradation efficiency (4.24 percent and 4.11 percent, respectively).

## Introduction

Everybody of water gradually transitions toward eutrophication over time as a result of rapid population growth, the expansion of agriculture and industry, dwindling freshwater resources, forest degradation, soil erosion, climate change, and repeated droughts. An essential result of this process is a general decrease in the availability of water for use and an increase in the significance of lakes and other water basins; as a result, many resources for socioeconomic development may be seriously compromised^1^. Algal blooms can be triggered by a decrease in water supply, a decrease in lake and reservoir depth, an increase in stagnation, an increase in nutrients from diverse sources, and a rise in temperature^2,3^. Numerous issues arise from algal substances in the water, including (1) pH, alkalinity, hardness, dissolved oxygen, and organic matter, (2) The increase in coagulation dose, (3) Physical indicators of water quality, such as color, flavor, odor, and cloudiness, to deteriorate as a result, (4) Filter blockage and decreased filter run, (5) Chlorine demand is rising, and by-products of disinfection are being produced, (6) Algae also cause other issues like forming a slimy and gelatinous layer, corrosive, and interference with other purification processes, (7) On direct contact, some types of algae can irritate the skin and trigger allergic reactions; However, different algae have been known to produce harmful toxins that are deadly to people and can even result in death in some extreme cases^4–9^. These issues can make filtered water unappealing and detrimental; on the other hand, they can also increase the price of drinking water purification by adding more chemicals, as well as increasing the workload for treatment plant workers^9^.

An important indicator used to describe biomass that uses light energy and is autotrophic is the concentration of chlorophyll-a. It is a crucial parameter reflecting the nutrient status of water bodies and can be used to estimate phytoplankton biomass and productivity. Due to their small-size, high mobility, low density, and negatively charged surface, algal cells in many existing water treatment plants cannot be removed or treated^5,10,12^. Algae from water sources are controlled using various physical, chemical, and biological techniques. Aeration, dissolved air flotation, filtration, skimming, mixing, membrane processes, ultraviolet, ultrasonic, electrolysis, and other related techniques are used to prevent harmful algae using the physical control method. Algal population management can be accomplished through biological processes like slow sand filters or activated sludge. The main chemical processes are coagulation, copper sulfate, activated carbon, nano particles, oxidation, hydrogen peroxide, and chlorination^13–19^. Most chemicals, however, are overpriced and too general, harming aquatic organisms that aren't their intended targets. Most toxic chemical types are not species-specific, which may harm the ecological balance. The potential for environmental harm from improper chemical application is lower than that from artificial mixing. The aeration technology may have drawbacks due to the high maintenance costs (labor costs) and energy consumption needed, and it does not kill the algae. Additionally, aquatic plants add a lot of oxidizing agents, which means that the disinfection by-products they produce are above the required level. The operational cost of algae removal in the water plant using membrane filtration, air flotation, and other techniques is high when the amount of algae in the water is high. The residual Al/Fe in treated water sometimes exceeds the upper limit of water standards, which poses a serious threat to human health, even though using chemicals to remove algae causes secondary pollution. But cyanobacteria oxidation can cause cell lysis. When intracellular organic matter (IOM) is released in large quantities, the water quality can suffer^10,20–22^.

In hydrodynamic cavitation (HC) reactors, voids are created as a result of pressure fluctuations that occur in the liquid as a result of passage through the constriction (such as a throttling valve, orifice plate, venturi, etc.)^23–25^. Cavitation is the formation and immediate implosion of cavities in a liquid what is subjected to rapid changes in pressure. The fluid's kinetic energy increases as it passes through the obstruction, at the expense of the local pressure. The liquid vaporizes and forms a series of cavities when the pressure at the mechanical stenosis's neck, or vena contracta drops below the liquid's vapor pressure. The cavities finally collapse when the pressure rises downstream of the mechanical stenosis. Cavity collapse causes the development of hotspots, the release of reactive free radicals, surface cleaning or erosion, and an improvement in mass transit. It has been hypothesized that in these circumstances, water molecules split into hydroxyl (^**∙**^OH) and hydrogen (^**∙**^H) radicals, which can attack and weaken the chemical makeup of the algal cell wall to the point of disintegration. During cavitation collapse, these localized hot spots have temperatures of about 5000 k, pressures of 1000 atmospheres, and lifetimes of a few microseconds. Another inactivation mechanism involves damage to the photosynthesis pathways^26–29^. Hydrodynamic cavitation harms algal cells by destroying gas vacuoles and cell walls, as well as reducing photosynthetic activity. A further mechanism of the hydrodynamic cavitation process is mentioned in the literature^20,30–32^. As thermal degradation. Many studies have demonstrated that the limited rate of oxidizing radical generation makes the degree of mineralization achieved by hydrodynamic cavitation alone insufficient. Combining HC with appropriate other advanced oxidant processes (AOPs) can help to increase the process efficiency and, as a result, the current work objectives in the development of hybrid treatment approaches^33–36^. Algal degradation is facilitated by a dual mechanism thanks to the combined use of HC, O_3_, and H_2_O_2_. The compound is degraded due to hydroxyl radicals produced during the direct attack of molecules of ozone and hydrogen peroxide. Additionally, the removal of the process's mass transfer restrictions was caused by high turbulence brought on by hydrodynamic cavitation^37–39^. The use of experimental design techniques, however, can be helpful to efficiently streamline the process and decrease the number of experiments. The Taguchi design approach is a popular experimental design technique for process modeling and evaluation. The aim of this approach is to improve the response variable, which is affected by several process parameters. Additionally, it guarantees effective process design performance. In recent years, Algal eutrophication has been a common occurrence at the Sanandaj Vahdat Dam in Iran. The water was then tested for flavor and aroma. Water users have expressed their disapproval and worries about the safety and quality of the water as a result. The goal of this study is to assess the effect of a combined system of hydrodynamic cavitation, ozone, and hydrogen peroxide on the removal of chlorophyll-a and organic substances in raw water. The distance of the orifice plate from the beginning of the cavitation tube was considered in this research, while this factor was not investigated in previous studies.

## Materials and methods

### pilot and materials

**Figure 1:**
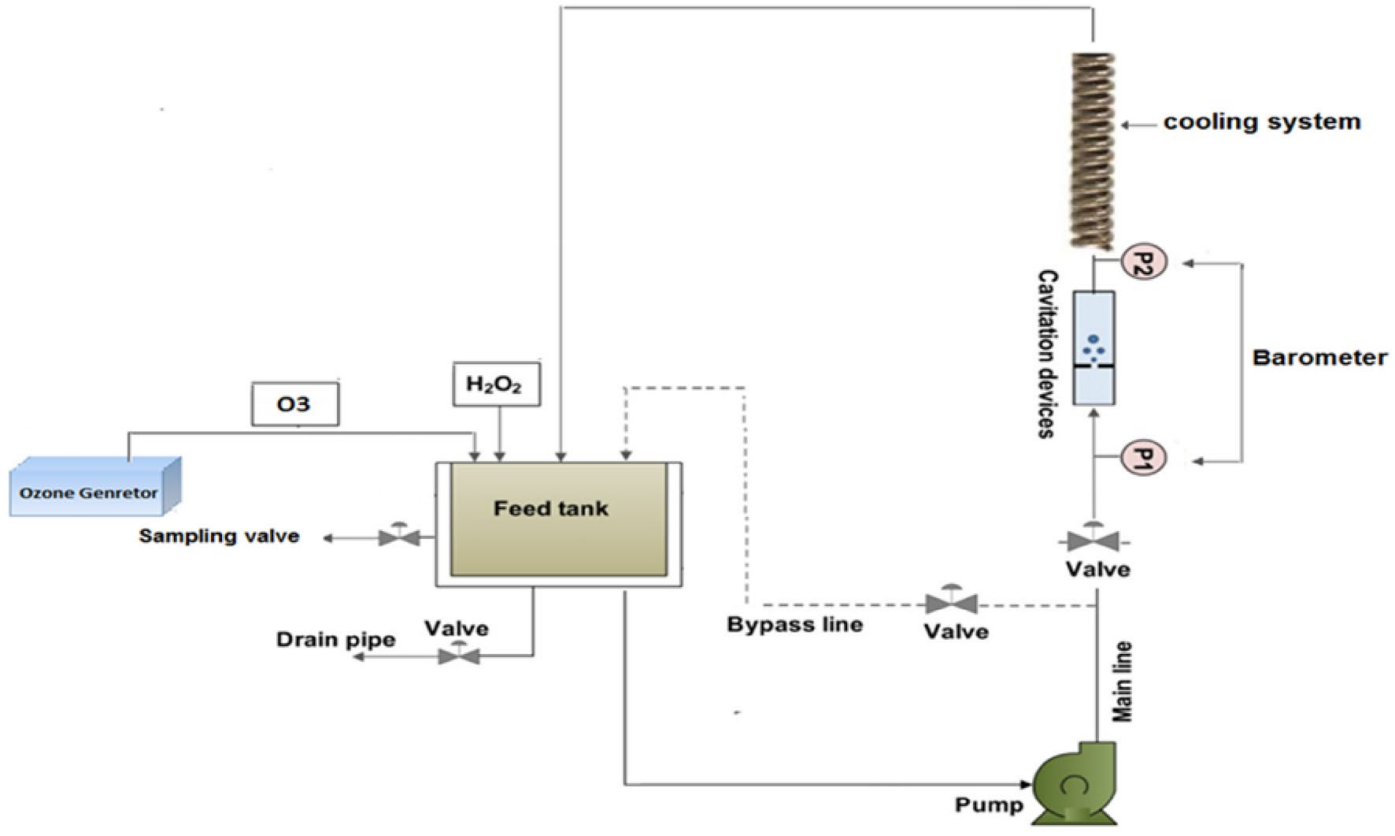
Schematic of the experimental setup.

This water was used for the pilot because it had the characteristics of the incoming water to the Sanandaj water treatment plant, which are variable (Table 1). The experimental setup for using ozone and hydrogen peroxide in conjunction with hydrodynamic cavitation is shown in Fig. 1. There is a jet flow loop and a 20-L polyethylene tank in it. The cooling water circulation system controls the temperature of the water in the reactor. A centrifugal pump of 2 HP (CB210), purchased from Electrogen, was used to pump the water flowing through the cavitation device through an attached 25 mm ID steel tube. When comparing orifices with the same cross-sectional flow area, multi-hole orifices produce more cavities than single-hole orifices; therefore, a 5-hole orifice plate (1 mm) was used. The pressure was measured using two manometers (model EN837, Dragon). The required ozone concentration was produced by an ozone generator (made by Pakzhi Company). During the experiment, the tank was filled with 35 percent w/v of analytical-grade hydrogen peroxide (H_2_O_2_) that was purchased from Merck in Germany. The TOC analyzer (Analytic, Jena, Germany) was used to perform the analysis. A spectrophotometer (Hach DR 4000U) and the method's standard instructions (plankton-10200) were used to analyze chlorophyll a, respectively^40^.

**Table 1:** Characteristics of the raw water used in the cavitation reactor.

Parameter	Mean value ± S.E (n = 48)	Min	Max
Chlorophyll a (μg/l)	8.25	3.5	17
TOC (mg/l)	5.75	3.2	6.68
pH	8.16	7.65	8.59
Tempreture	14	7.2	15.9

### Parameters

Seven parameters were chosen as the main parameters for the study of the hydrodynamic cavitation process, including pH (5, 7, 9), retention time (20, 60, 90 min), cavitation pressure (1, 3, 5 bar), flow rate (1, 3, 5 m^3^/h), the distance of the orifice from the beginning of the cavitation tube (25, 50, 75 cm), ozone concentration (0, 2, 3 g/h), and hydrogen peroxide concentration (0, 1, 2 g/l). *Chlorophyll a* (algae index) and *TOC* were chosen as the two factors.

### Designation and optimization of experiment

The Taguchi method is a potent approach to problem-solving that raises productivity, yield, and process performance. Through the systematic use of the statistical design of experiments, also known as robust design, Taguchi's main goal is to reduce variability around the target value of product properties. Taguchi explained that the framework could be seen in three main components: (1) Systems Design (it may include the best fusion of materials and procedures), (2) Design of the parameters (which includes the best set of guidelines for the recognized design components), (3) Tolerance Design: Look at the factors that play a significant role in product quality^41,42^. The required variation in the design is then provided by tolerance limits that are then identified. By evaluating the impact of variables on removal efficiency (response), experimental results can be analyzed using the signal-to-noise ratio (S/N). A dimensionless parameter (metric) known as the signal-to-noise ratio measures the discrepancy between a response and the desired value. Three types of signal-to-noise analysis are commonly used. Lower is better (LB), nominally better (NB), and higher is better (HB), respectively. A larger S/N was chosen since the primary objective of optimization in this study was to achieve the highest removal efficiency. Thus, for the larger one, the S/N ratio is a better criterion in the Eq. (1) is:

1 $$\frac{S}{N}=-10\mathrm{log}10\left[\frac{1}{n}\sum_{i=1}^{n}\frac{1}{EF2}\right],$$

2 $$\mathrm{EF \% }= \frac{C1-C2}{\mathrm{C}0}\times 100.$$

The n represents the number of repetitions of the experiment and the EF represents the outcomes of the measurements. The removal efficiency of chlorophyll a and TOC is obtained from Eq. (2), where C1 and C2 are the initial and equilibrium concentrations of pollutants (chlorophyll a and TOC, respectively). After designing the experiment using the Taguchi method for the seven parameters, 27 proposed run steps. Tables 2 and 3 provide specifics about each experiment. Every experiment was run twice, put into the model, and then analyzed. Here, the analysis of the statistical mean value approach (ANOM) is utilized to produce ideal conditions^43,44^. First, the average of the S/N ratio of each factor at a given level should be calculated (Eq. 3).

3 $$(\mathrm{M})\mathrm{Factor}=\mathrm{I}=\frac{1}{n }\sum_{j=1}^{n Ii}[(\frac{S}{N}) \mathrm{Factor}=\mathrm{I}]\mathrm{ j},$$

where [(S/N) Factor = I] is the S/N ratio of Factor I in Level i, I is the mean S/N ratio of Factor I at Level i, nIi denotes the number of instances of Factor I in Level I, and the order of appearance in Tables 4 and 5 is the jth. Each controllable factor's.

**Table 2:** The runs of the Taguchi design experiment and the outcomes in chlorophyll a are referred to by S/N values.

Row	pH	T (min)	P (bar)	Q (m^3^/h)	D (m)	O_3_ (g/h)	H_2_O_2_ (g/l)	EF_1_ (Chlorophyll a) %	EF_2_ (Chlorophyll a) %	S/N (Chlorophyll a)
1	5	20	1	1	25	0	0	42.84	45.06	32.8592
2	5	60	3	3	25	0	0	63.2	64.85	36.1236
3	5	90	5	5	25	0	0	85.08	83.12	38.4959
4	7	20	3	5	25	2	2	55.2	56.1	34.9015
5	7	60	5	1	25	2	2	98	98.61	**39.85**
6	7	90	1	3	25	2	2	71.19	70.46	37.0007
7	9	20	5	3	25	3	1	75.5	76.62	37.6220
8	9	60	1	5	25	3	1	49	47.92	33.7077
9	9	90	3	1	25	3	1	88	86.7	38.8253
10	5	20	1	1	50	2	1	42.04	44.08	32.7177
11	5	60	3	3	50	2	1	66.24	65.62	36.3817
12	5	90	5	5	50	2	1	86.57	87.87	38.8123
13	7	20	3	5	50	3	0	35.39	34.95	30.9234
14	7	60	5	1	50	3	0	84.16	82.83	38.4389
15	7	90	1	3	50	3	0	51.63	52.21	34.3067
16	9	20	5	3	50	0	2	53.88	53.64	34.6060
17	9	60	1	5	50	0	2	28	29.61	29.1878
18	9	90	3	1	50	0	2	65.6	65.1	36.2983
19	5	20	1	1	75	3	2	42.72	43.66	32.7057
20	5	60	3	3	75	3	2	67.16	68	36.5964
21	5	90	5	5	75	3	2	90	89.72	39.0713
22	7	20	3	5	75	0	1	17.3	17.3	24.7559
23	7	60	5	1	75	0	1	65.5	64.02	36.2261
24	7	90	1	3	75	0	1	34.84	35.67	30.9481
25	9	20	5	3	75	2	0	43.3	41.38	32.5350
26	9	60	1	5	75	2	0	18.00	18.00	25.1055
27	9	90	3	1	75	2	0	57.05	54.26	34.9109

Significant values are in bold.

**Table 3:** The runs of the Taguchi design experiment and the outcomes in *TOC* are referred to by S/N values.

Row	pH	T (min)	P (bar)	Q (m^3^/h)	D (m)	O_3_ (g/h)	H_2_O_2_ (g/l)	EF1 (TOC)%	EF2 (TOC)%	S/N (TOC)
1	5	20	1	1	25	0	0	20.5	21.5	26.4444
2	5	60	3	3	25	0	0	42	43	32.5678
3	5	90	5	5	25	0	0	67.5	66.1	36.4955
4	7	20	3	5	25	2	2	33	30.62	30.0485
5	7	60	5	1	25	2	2	79.5	81	**38.0889**
6	7	90	1	3	25	2	2	53	52	34.4032
7	9	20	5	3	25	3	1	49.8	51.19	34.0658
8	9	60	1	5	25	3	1	29	29.8	29.3669
9	9	90	3	1	25	3	1	71.9	70.52	37.0496
10	5	20	1	1	50	2	1	26	27	28.4649
11	5	60	3	3	50	2	1	46.2	47.4	33.4049
12	5	90	5	5	50	2	1	72.65	70.75	37.1104
13	7	20	3	5	50	3	0	17	17.5	24.7358
14	7	60	5	1	50	3	0	64.33	65.72	36.2583
15	7	90	1	3	50	3	0	37	38	31.4806
16	9	20	5	3	50	0	2	31.4	30	29.7428
17	9	60	1	5	50	0	2	8.5	9.3	18.9878
18	9	90	3	1	50	0	2	50	50	33.9794
19	5	20	1	1	75	3	2	33.8	32.8	30.4489
20	5	60	3	3	75	3	2	53.25	54.52	34.6318
21	5	90	5	5	75	3	2	78	79	37.8974
22	7	20	3	5	75	0	1	2.8	3.15	9.53879
23	7	60	5	1	75	0	1	51.05	49.34	34.0141
24	7	90	1	3	75	0	1	22	22	26.8485
25	9	20	5	3	75	2	0	24.2	25.75	27.9240
26	9	60	1	5	75	2	0	3	3	9.5424
27	9	90	3	1	75	2	0	43.7	45.5	32.9867

Significant values are in bold.

**Table 4:** Results of the ANOM analysis to determine the best conditions for *chlorophyll a*.

Factor/level	j = 1	j = 2	j = 3	j = 4	j = 5	j = 6	j = 7	J = 8	j = 9	M (level factor)
pH(1)	32.85	36.12	38.49	32.71	36.38	38.81	32.70	36.59	39.07	**65.45**
pH(2)	34.90	39.93	37	30.92	38.43	34.3	24.75	36.22	30.94	57.07
pH(3)	37.62	33.7	38.82	34.60	29.18	36.29	32.53	25.10	34.91	52.85
Time(1)	32.85	32.71	32.70	34.90	30.92	24.75	37.62	34.60	32.53	32.63
Time(2)	36.12	36.38	36.59	39.93	38.43	36.22	33.70	29.18	25.10	34.63
Time(3)	38.49	38.81	39.07	37	34.30	30.94	38.82	36.29	34.91	**36.52**
Pressure(1)	25.10	29.18	30.94	32.70	32.72	32.85	33.70	34.30	37	32.06
Pressure(2)	24.75	30.92	34.9	34.91	36.12	36.29	36.38	36.59	38.82	34.41
Pressure(3)	32.53	34.6	36.22	37.62	38.43	38.49	38.81	39.07	39.93	**37.31**
Flow (1)	32.70	32.71	32.85	34.91	36.29	38.82	36.22	38.43	39.93	**35.88**
Flow (2)	30.94	34.3	37	36.12	36.38	36.59	32.53	34.60	37.62	35.12
Flow (3)	25.10	29.18	33.7	24.75	30.92	34.9	38.49	38.81	39.07	32.77
Distance(1)	32.85	38.82	39.93	37	36.12	37.62	33.7	34.9	38.49	**36.61**
Distance(2)	32.71	36.29	38.43	34.3	36.38	34.6	29.18	30.92	38.81	34.63
Distance(3)	32.70	34.91	36.22	30.94	36.59	32.53	25.10	24.75	39.07	32.54
O_3_(1)	32.85	36.12	38.49	36.29	34.6	29.18	36.22	30.94	24.75	33.28
O_3_(2)	39.93	37	34.9	32.71	36.38	38.81	34.91	32.53	25.10	34.70
O_3_(3)	38.82	37.62	33.7	38.43	34.3	30.92	32.7	36.59	39.07	**35.8**
H_2_O_2_(1)	32.85	36.12	38.49	34.91	32.53	25.10	38.43	34.3	30.92	33.74
H_2_O_2_(2)	36.2261	30.94	24.75	32.71	36.38	38.81	38.82	37.62	33.70	34.44
H_2_O_2_(3)	36.29	34.6	29.18	39.93	37	34.90	32.70	36.59	39.07	**35.59**

Significant values are in bold.

**Table 5:** Results of the ANOM analysis to determine the best conditions *TOC.*

Factor/level	j = 1	j = 2	j = 3	j = 4	j = 5	j = 6	j = 7	J = 8	j = 9	M (level factor)
PH(1)	17.50	29	34.56	21.21	29.99	35.08	24.19	31.68	36.09	**28.82**
PH(2)	25.48	36.31	31.95	13.97	34.18	27.08	9.54	30.75	17.38	25.19
PH(3)	30.42	21.43	34.51	23.80	11.82	30.88	20.25	9.54	29.39	23.56
Time(1)	17.5	21.21	24.19	25.48	13.97	9.54	30.42	23.8	20.25	20.71
Time(2)	29	29.99	31.68	36.31	34.18	30.75	21.43	11.82	9.54	26.08
Time(3)	34.56	35.08	36.09	31.95	27.08	17.38	34.51	30.88	29.39	**30.77**
Pressure(1)	17.50	21.21	24.19	21.43	11.82	9.54	31.95	27.08	17.38	20.24
Pressure(2)	25.48	13.97	9.54	29	29.99	31.68	34.51	30.88	29.39	26.05
Pressure(3)	30.42	23.80	20.25	36.31	34.18	30.75	34.56	35.08	36.09	**31.28**
Flow (1)	17.50	21.21	24.19	34.51	30.88	29.39	36.31	34.18	30.75	**28.77**
Flow (2)	31.9539	27.08	17.38	29	29.99	31.68	30.42	23.8	20.25	26.83
Flow (3)	21.43	11.82	9.54	25.48	13.97	9.54	34.56	35.08	36.09	21.95
Distance(1)	17.50	34.51	36.31	31.95	29	30.42	21.43	25.48	34.56	**29.02**
Distance(2)	21.2140	30.88	34.18	27.08	29.99	23.8	11.82	13.97	35.08	25.34
Distance(3)	24.19	29.39	30.75	17.38	31.68	20.25	9.54	9.54	36.09	23.21
O_3_(1)	17.50	29	34.56	30.88	23.8	11.82	30.75	17.38	9.54	22.81
O_3_(2)	36.31	31.95	25.48	21.21	29.99	35.08	29.39	20.25	9.54	26.58
O_3_(3)	34.51	30.42	21.43	34.18	27.08	13.97	24.19	31.68	36.09	**28.18**
H_2_O_2_(1)	17.50	29	34.56	29.39	20.25	9.54	34.18	27.08	13.97	23.95
H_2_O_2_(2)	30.75	17.38	9.54	21.21	29.99	35.08	34.51	30.42	21.43	25.60
H_2_O_2_(3)	30.88	23.8	11.82	36.31	31.95	25.48	24.19	31.68	36.09	**28.03**

Significant values are in bold.

Impact on the separation of *Chlorophyll a* and *TOC* is also examined using the statistical technique of analysis of variance (ANOVA). The percentage contribution of each factor, RF, is given by Eq. (4):

4 $$ {\text{RF}} = {\text{SSF }}{-} \, ({\text{DF}} \times {\text{VER}}) \times {1}00/{\text{SS}}_{{\text{T}}} . $$

Each parameter's degree of freedom (DF) is one number less than the number of levels in the factor, which in this study are two.

The total sum of squares, SST, is given by Eq. (5)

5 $$ {\text{SS}}_{{\text{T}}} = \mathop \sum \limits_{j = 1}^{m} \left( {\mathop \sum \limits_{j = 1}^{n} EFi2} \right) - {\text{ mn}}\left( {{\text{EF}}_{{\text{T}}} } \right)^{{2}} . $$

Equation (6) is used to determine the value of EF_T_. Where m (27 experiments) and n (two repetitions) denote the number of experiments and number of experiments, respectively.

6 $$ {\text{EF}}_{{\text{T}}} = \mathop \sum \limits_{j = 1}^{m} \left( {\mathop \sum \limits_{j = 1}^{n} EFi} \right)/{\text{mn}}{.} $$

The sum of factor squares (SSF) is calculated using Eq. (7):

7 $$ {\text{SS}}_{{\text{F}}} = {\text{ mn}}/{\text{L}}\mathop \sum \limits_{K = 1}^{m} \left( {{\text{E}}\overline{{{\text{F}}_{{\text{K}}}^{{\text{F}}} }} - {\text{E}}\overline{{{\text{F}}_{{\text{T}}} }} } \right)^{{2}} . $$

EF_k_^F^ the average of the measurement results of a certain factor in the kth level.

In addition, the error variance, VEr, is given by Eq. (8):

8 $$ {\text{V}}_{{{\text{ER}}}} = {\text{ SS}}_{{\text{T}}} - \mathop \sum \limits_{{{\text{F}} = {\text{A}}}}^{{\text{E}}} {\text{SSF/m}}\left( {{\text{n}} - {1}} \right). $$

## Results and discussions

### Optimization

For each test condition, the S/N ratio is calculated in Tables 2 and 3. The maximum signal-to-noise ratio among the 27 tests is indicated in bold type in these tables. According to Tables 4 and 5, the ideal conditions for removing *TOC* and *chlorophyll a* are as follows: pH = 5, retention time = 90 min, cavitation pressure = 5 bar, water flow = 1 m^3^/h, orifice plate distance = 25 cm, ozone value = 3 g/h, and H_2_O_2_ concentration = 2 g/l. The confirmation experiment was carried out under the aforementioned ideal circumstances, the EF of *chlorophyll a* and *TOC* were measured, and the S/N ratio was computed. Table 6 shows the efficiency difference between the optimal and test 5 conditions were about 1.7 percent for *chlorophyll a*, and it was about 19.75 percent for *TOC*. Due to the difference in the 30-min retention time and the requirement to adjust the pH, Run 5 is more cost-effective than the ideal state when it comes to the consumption of ozone and hydrogen peroxide.

**Table 6:** The optimum conditions for *chlorophyll a* and *TOC* removal.

Parameters	pH	Time	Pressure	Flow	Distance	O_3_	H_2_O_2_	EF_1_	EF_2_	S/N
Test 5 for chlorophyll a removal	7	60	5	1	25	2	2	98	98.61	**39.85**
Optimization condition for chlorophyll a removal	5	90	5	1	25	3	2	100	100	40
Test 5 for TOC removal	7	60	5	1	25	2	2	79.5	81	**38.08**
Optimization condition for TOC removal	5	90	5	1	25	3	2	100	100	40

Significant values are in bold.

### Effect of studied factors

**Figure 2:**
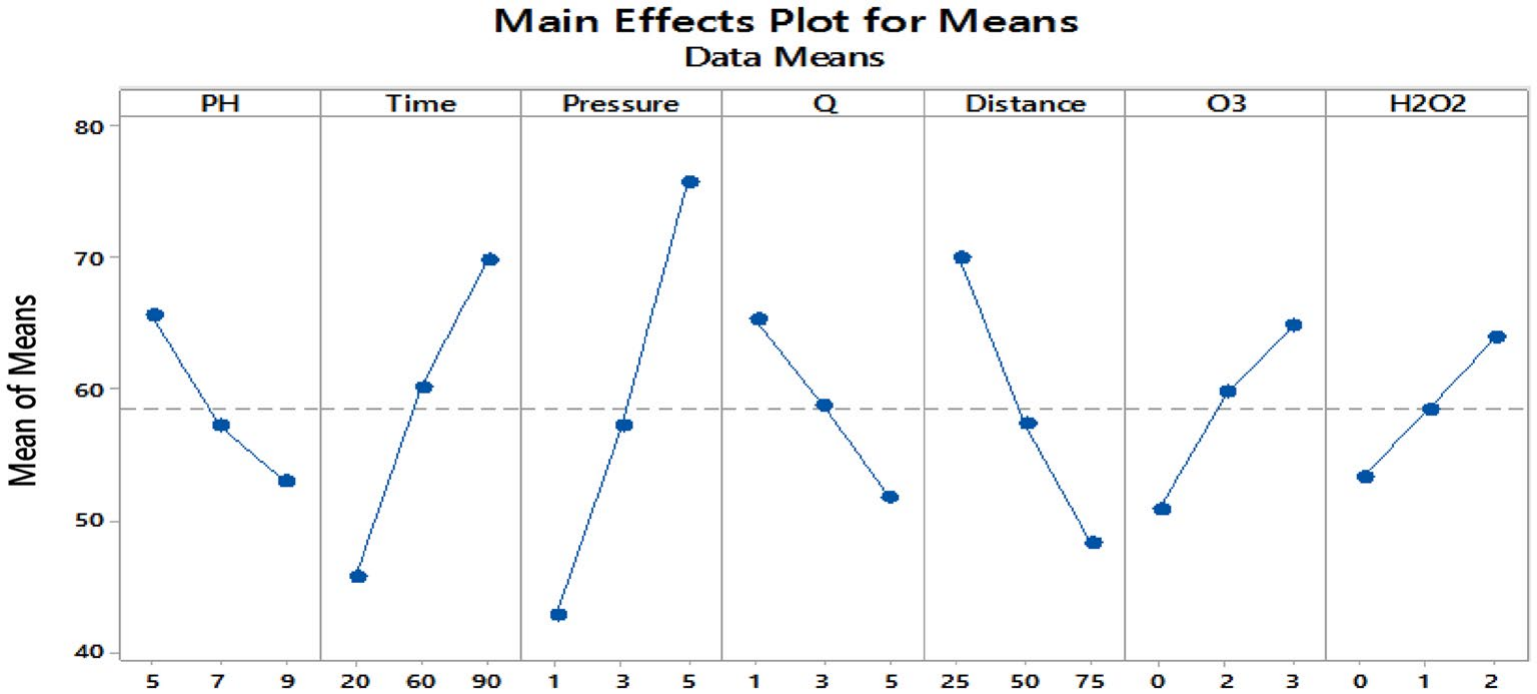
Main effect diagram for means of *chlorophyll a* removal.

**Figure 3:**
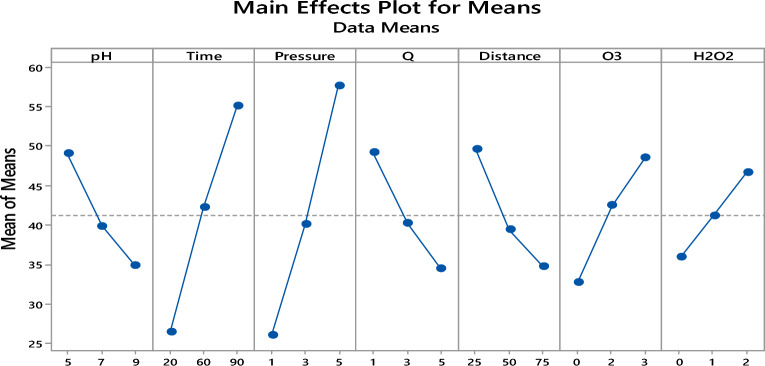
Main effect diagram for means of *TOC* removal.

Influence of factors under investigation The production of hydroxyl radicals (Eq. 1) is the primary mechanism of pollutant degradation by the hydrodynamic cavitation process^9–11^. Hydrodynamic cavitation produces hydroxyl radicals, and the amount and rate of formation are influenced by variables and reactor conditions. The main influences on the removal of *TOC* and *chlorophyll a* are depicted in Figs. 2 and 3, respectively. According to these figures, hydrogen peroxide has the most negligible impact on cavitation pressure and a greater impact on cavitation pressure.

9 $$ {\text{H}}_{2} {\text{O}}\xrightarrow{{{\text{HC}}}}{}^{ \cdot }{\text{H }} + ^{ \cdot } {\text{OH,}} $$

10 $$^{ \cdot } {\text{OH}}^{{}} +^{ \cdot } {\text{OH}} \to {\text{H}}_{{2}} {\text{O}}_{{2}} , $$

11 $$ {\text{Pollutants }} +^{ \cdot } {\text{OH}} \to {\text{CO}}_{{2}} + {\text{ H}}_{{2}} {\text{O }} + {\text{Intermediate}}\,{\text{products}}\,{\text{of}}\,{\text{degradation}}\left( {{45} - { 46}} \right). $$

#### pH effects

**Figure 4:**
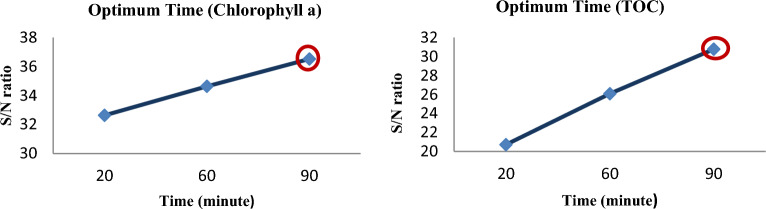
The impact of Retention Time on removal of *Chlorophyll a* and *TOC.*

**Figure 5:**
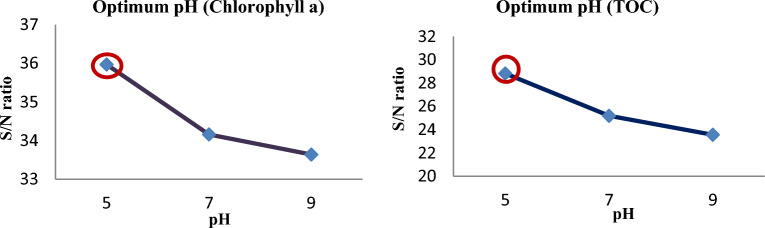
The impact of pH on the removal of *Chlorophyll a* and *TOC.*

The pH of the water is an important parameter in determining the extent of the degradation of the organic pollutants by the HC process. Figure 4 shows the percentage reduction in chlorophyll a and TOC of the water sample as the pH changed. It was discovered that as the pH increased from 5 to 9, the percentage reduction also started to decrease (Fig. 5). Thus, based on the findings of this study, cavitation produces the largest degradation when operating in an acidic environment, and less severe degradation when used in an alkaline environment. Algae in water sources usually have a negative charge (zeta potential), and the ideal values also depend on the specific compounds' pKa during processing. The zeta potential (ZP) of the algal cells needs to be destabilized to improve the removal of algal cells during water purification. In acidic media, the generation of %^**∙**^OH radicals is preferred and also has a higher oxidizing capacity. "Additionally, the recombination probability of the ^**∙**^OH radicals is low, resulting in a higher number of ^**∙**^OH radicals in the solution to degrade the target contaminant. With an increase in pH, the recombination of **∙**OH radicals takes place, reducing their ability to degrade the target contaminant. Numerous studies support this finding, highlighting that as pH increases, the removal efficiency of parameters decreases." Several studies have supported this as the pH increases, the removal efficiency of the parameters decreases^47–52^.

#### Time effect

The cavitation flow, ozone, and hydrogen peroxide injection all increase with increasing cavitation time, which also accelerates the rate at which free radicals are produced and pollutants are broken down. The production of free radicals and the rate at which organic matter and chlorophyll decompose also rise as the cavitation time increases along with the cavitation flow, ozone injection, and hydrogen peroxide injection. The Chlorophyll a and TOC values of the cavitated liquid are likely to continue to decline as the processing time increases, but this is also likely to result in a higher energy need for the procedure. This figure illustrates the relationship between the contact time and the removal efficiency of chlorophyll a and TOC. Other studies have supported these results^50–53^.

#### Pressure effects

**Figure 6:**
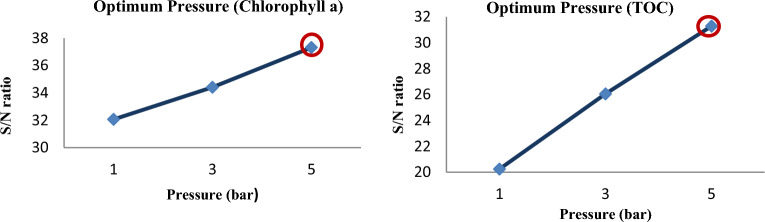
The impact of Pressure on the removal of *Chlorophyll a* and *TOC.*

As the liquid passes through strictures like the orifice, the pressure at the vena contract falls below the vapor pressure of the liquid, causing the liquid to flash and produce a series of bubbles that later burst when pressure is restored downstream of the stricture. The photosynthetic system and membrane structure of algal cells can be damaged by high pressure and the hydroxyl radicals that are produced during the HC process. Thus, cavitation effectiveness and the generation of free radicals are influenced by pressure. Results for how inlet pressure affected this study's findings. Figure 6 illustrates how changes in inlet pressure result in an increase in the percentage of chlorophyll a and TOC removal. This is because more cavities are created as the inlet pressure rises, which leads to an increase in the percentage of OH radical formation and organic degradation^20,54–56^. Jadhav et al. demonstrated Imidacloprid removal using a cavitation device combined with oxidants and reported that increasing the inlet pressure from 5 to 15 bar increased the degradation efficiency of Imidacloprid^57^. The research's findings corroborated earlier studies that found cavitation efficiency rises with pressure up to 5 bar^58,60^. Increasing the cavitation pressure leads to increase in the velocity of the fluid at the orifice hole and then, more cavitation bubbles are generated and the intensity of cavitation also increases, thereby leading to the formation of more %^**∙**^OH radicals and more degradation of the organic content^49^.

#### Flow effect

**Figure 7:**
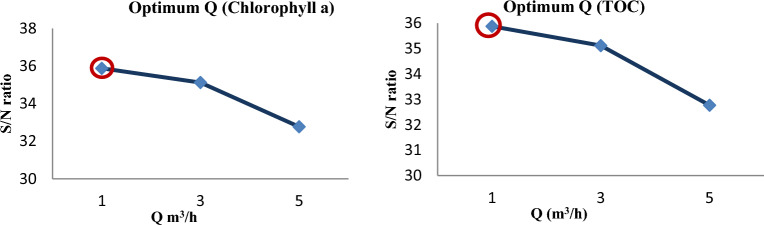
The impact of Flow on the removal of *Chlorophyll a* and *TOC.*

The ability of hydrodynamic cavitation reactors to process contaminants more than once during a single operation is the desired benefit. Under other studies, decomposition is made simple and the number of free radicals produced per pollutant unit rises when the flow rate is reduced^60,61^. Figure 7 also illustrates how this study's findings adjust with those of other studies.

#### Distance effect

**Figure 8:**
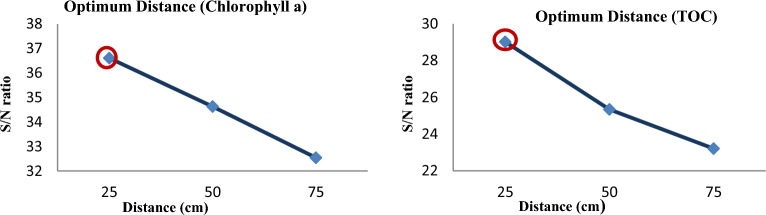
The impact of Distance on the removal of *Chlorophyll a* and *TOC.*

The removal efficiency of chlorophyll a and TOC increased with decreasing orifice plate distance from the cavitation tube, as shown by the study's findings (Fig. 8). This might be connected to a hole in the low-pressure region having a longer retention time. Additionally, holes and heat are produced on the orifice plate when the water that has been infused with energy and pressure from the pump strikes it. The geometry of the cavitation devices affects the hydrodynamic cavitation reactor's efficiency. So, the geometry of cavitation devices is dependent on how long the cavity remains in the low-pressure region. Thus, it is likely that the cause of the increase in algae removal efficiency at distances near the orifice plate with the primary cavitation tube is the increase in the cavitation time of a cavity in a low-pressure region. Since, over a short distance, the incident energy and pressure change are increased and the distance between the orifice plate and the cavitation tube's starting point is decreased, the cavitation intensity is probably increased. Thus, the effectiveness of hydrodynamic cavitation depends on the orifice plate's position^20,59,63–66^.

#### Ozone effect

**Figure 9:**
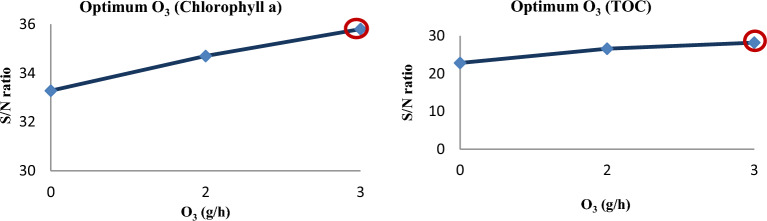
The impact of O_3_ on the removal of *Chlorophyll a* and *TOC.*

Ozone-assisted hydrodynamic cavitation can be used to increase the oxidation intensity of pollutants while reducing ozone consumption. In hydrodynamic cavitation by ozone, it has been confirmed that the oxidation of contaminants occurs immediately after the injection of ozone. The combined operation of ozone and cavitation ensures that, in addition to being directly attacked by ozone, pollutants are also decomposed by hydroxyl radicals. Also, the local turbulence generated by cavitation contributes to the mass transfer of ozone from the gas phase to the bulk, so the rate of reaction of ozone with pollutant molecules is not very significant due to high mass transfer resistances in water. In addition to this, ozone dissociates in the presence of cavitation and generates atomic oxygen (^**∙**^O), which further reacts with water molecules to generate highly reactive ^**∙**^OH radicals. The combined effect of adding ozone and HC improved the degradation efficiency in both pH ranges (acidity and alkalinity) compared to HC treatment alone or ozone treatment alone. This is because the combined operation of ozonation and HC renders a synergistic effect^52,67–70^. It can be seen in Fig. 9 that the effect of ozone on removing chlorophyll is greater than that of TOC. The reason for this is probably the destruction of algal gas vacuoles by ozone. Also shown in this graph, the pollutant removal efficiency increases with the increase in ozone concentration. The reason is the increase in free radical production per pollutant, which is consistent with previous studies^70,71^.

12 $$ {\text{H}}_{{2}} {\text{O}}\mathop{\longrightarrow}\limits^{{{\text{HC}}}}{\text{H}}^{ \cdot } + {\text{ OH}}^{ \cdot } , $$

13 $$ {\text{O}}_{{3}} \mathop{\longrightarrow}\limits^{{{\text{HC}}}}{\text{O}}_{{2}} + {\text{ O }}\left( {^{{3}} {\text{P}}} \right), $$

14 $$ {\text{O }}\left( {^{{3}} {\text{P}}} \right) \, + {\text{ H}}_{{2}} {\text{O}} \to {2}^{ \cdot } {\text{OH,}} $$

15 $$ {\text{Algae }} +^{ \cdot } {\text{OH}} \to {\text{CO}}_{{2}} + {\text{ H}}_{{2}} {\text{O }} + {\text{Intermediate}}\,{\text{products}}\,{\text{of}}\,{\text{degradation }}\left( {{2}0} \right). $$

#### Hydrogen peroxide effect

**Figure 10:**
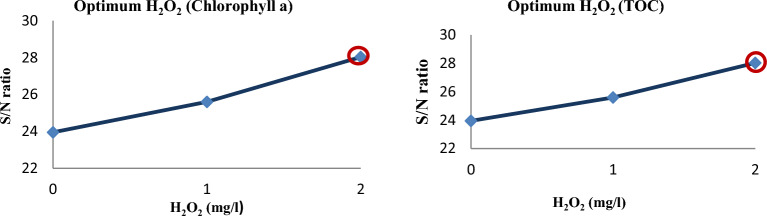
The impact of *H*_*2*_*O*_*2*_ on the removal of *Chlorophyll* a and *TOC.*

With increasing H_2_O_2_ dosages (0–2 g/l), the results are shown in Fig. 10 for the degradation of chlorophyll a and TOC. The creation and then attack of hydroxyl radicals is a critical mechanism that aids in the degradation of pollutants by HC-H_2_O_2_ (Eqs. 16–18). Because hydrogen peroxide accelerates the oxidation of chlorophyll a and TOC by producing ^**∙**^OH, the removal efficiency of chlorophyll a and TOC increases with increased hydrogen peroxide concentration. In the presence of HC and H_2_O_2_, owing to the high pressure and temperature conditions created by cavitation, the dissociation of H_2_O_2_ and water resulted in numerous hydroxyl H_2_O_2_ loading increases. H2O2 was constantly dissociated under cavitation, resulting in an enhanced formation of hydroxyl radicals. H_2_O_2_ enhances the hydroxyl radical-induced degradation process by acting as a more abundant source of these hydroxyl radicals. A similar study of the treatment of actual industrial wastewater effluent has also observed that the efficacy of hydrodynamic cavitation is enhanced appreciably by using it in combination with H_2_O_2_. It was found that the extent of TOC reduction increased with an increase in the loading of H_2_O_2_. Other related studies have supported the findings^70,72–76^.

16 $$ {\text{H}}_{{2}} {\text{O}}_{{2}} \to {2}^{ \cdot } {\text{OH,}} $$

17 $$  {\text{H}}_{2} {\text{O}}\xrightarrow{{{\text{HC}}}}{}^{ \cdot }{\text{H }} + {\text{ OH}}^{ \cdot } ,  $$

18 $$ {\text{Algae }} +^{ \cdot } {\text{OH}} \to {\text{CO}}_{{2}} + {\text{ H}}_{{2}} {\text{O }} + {\text{Intermediate}}\,{\text{products}}\,{\text{of}}\,{\text{ degradation}}{.} $$

### Percentage of contribution

First, RE_k_^F^ is obtained and shown in Tables 7 and 8. RE_k_^F^ is the mean value of the measured results for the factor at the kth level. By replacing RE_k_^F^ and RE_T_ (*Chlorophyll a*: 58.45 and *TOC*: 41.28) into Eq. (7) the factorial sum of squares,SSF, for each factor was calculated for each factor and determined in Table 9. Using Eq. (5), the total sum of squares, SS_T_, was calculated. By changing SSF and SS_T_ in Eq. (8), the error variance, V_ER_, was obtained. Finally, by substituting SS_F_, SS_T_, V_ER_, and DOF_F_ in Eq. (4) to determine the percentage contribution of each factor, RF, the results are shown in Table 9.

**Table 7:** The mean of the measurement results for a particular factor at the k_th_ level and the mean of the total RE of *chlorophyll a*.

Parameter	Level	PRE_k_^pH^	PRE _k_^Time^	PRE _k_^Prssure^	PRE _k_^Flow^	PRE _k_^Distance^	PRE _k_^O3^	PRE _k_^H2O2^	PRE_T_
Chlorophyll a	1	65.45	45.61	42.62	65.14	70	50.8	53.18	58.45
	2	57.07	60.04	57.09	58.62	57.2	59.78	58.39	
	3	52.85	69.72	75.65	51.61	48.21	64.79	63.79

**Table 8:** The mean of the measurement results for a particular factor at the kth level and the mean of the total RE of *TOC.*

Parameter	Level	PRE_k_^pH^	PRE _k_^Time^	PRE _k_^Prssure^	PRE _k_^Flow^	PRE _k_^Distance^	PRE _k_^O3^	PRE _k_^H2O2^	PRE_T_
TOC	1	49	26.55	26.01	49.08	49.55	32.78	35.87	41.28
	2	39.94	42.21	40.11	40.11	39.37	38	41.25	
	3	35.02	54.97	57.61	34.48	34.82	48.5	46.65

**Table 9:** Determining the percentage contribution of each factor in the removal of *Chlorophyll a* and *TOC.*

Pollutants	Factor	DOF_F_	SS_F_	SS_T_	RF%	V_ER_
Chlorophyll a	pH	2	1480.68	25,540.22	5.79	1.29
	Time	2	5299.25		20.74	
	Pressure	2	9868.86		38.64	
	Flow	2	1648.16		6.45	
	Distance	2	4316.71		16.9	
	O_3_	2	1805.5		7.06	
	H_2_O_2_	2	1083.2		4.24	
TOC	pH	2	1810.08	25,400.27	7.12	2.5
	Time	2	7294.42		28.71	
	Pressure	2	9021.58		35.5	
	Flow	2	1951.92		7.68	
	Distance	2	2047.7		8.06	
	O_3_	2	2432.31		9.57	
	H_2_O_2_	2	1045.76		4.11	

## Conclusion

- Hydrodynamic cavitation systems have been recognized as a new form of multiphase reactors capable of producing favorable oxidation, including localized hotspots, turbulence, and reactive free radicals within the system. In this study, chlorophyll a and TOC were removed from water using a combination of hydrodynamic cavitation, ozone, and hydrogen peroxide. The ideal conditions for removing chlorophyll a and TOC are as follows: cavitation pressure of 5 bar, retention time of 90 min, pH: 5, flow of 1 m^3^/h, distance from the orifice of 25 cm, ozone of 3 g/h, and hydrogen peroxide of 2 g/l. According to the percentage contributions of each factor, cavitation pressure was identified as the factor that was most effective in the degradation of TOC and chlorophyll a (38.64 percent and 35.5 percent, respectively). H_2_O_2_ was found to have the least impact on degradation efficiency (4.24 percent and 4.11 percent, respectively). The issues and future research directions that merit careful consideration are listed below in light of the study's findings.
- The orifice plate's distance from the cavitation tube's beginning was considered in this study, but it was not in earlier ones. In this study, distances of 25, 50, and 75 cm were assessed; however, future studies may assess extra distances.
- The addition of ozone increased the effectiveness of removing pollutants while decreasing the amount of time needed to achieve this result. The main drawback of using ozone alone in water treatment plants is mass transfer, but hydrodynamic cavitation can increase the mass transfer of ozone from a gaseous phase to water.
- In this study, the synergistic effect of combining hydrodynamic cavitation with hydrogen peroxide and ozone oxidants was minimal. Changes in the organic load and algal input to the sewage treatment plant may be to blame for this (Table 1). This is so that the research could be done using actual raw water that entered the Sanandaj treatment facility.
- Throughout this study, several parameters, including a load of organic matter, rainfall, the temperature of the inlet water, and the load of algae, changed, which had an impact on how effectively the algae were removed.
- Due to its high efficiency in destroying microalgae, hydrodynamic cavitation has a lot of potential for treating nutrient-rich waters. Because it doesn't produce secondary pollution, hydrodynamic cavitation is also a sustainable abatement technique.

## Data Availability

Contains data required for analysis in manuscript. The corresponding author is willing to clarify the data and will provide all necessary datasets according to the request.
